# CD45RA, CD8β, and IFNγ Are Potential Immune Biomarkers of Human Cognitive Function

**DOI:** 10.3389/fimmu.2020.592656

**Published:** 2020-11-25

**Authors:** André J. Esgalhado, Débora Reste-Ferreira, Stephanie E. Albino, Adriana Sousa, Ana Paula Amaral, António Martinho, Isabel T. Oliveira, Ignacio Verde, Olga Lourenço, Ana M. Fonseca, Elsa M. Cardoso, Fernando A. Arosa

**Affiliations:** ^1^ CICS-UBI, Health Sciences Research Centre, University of Beira Interior, Covilhã, Portugal; ^2^ Molecular Genetics Laboratory, Coimbra Blood and Transplantation Center, Coimbra, Portugal; ^3^ C4-UBI, Cloud Computing Competence Centre, University of Beira Interior, Covilhã, Portugal; ^4^ Faculty of Health Sciences, University of Beira Interior, Covilhã, Portugal; ^5^ IPG, Guarda Polytechnic Institute, Guarda, Portugal

**Keywords:** effector-memory CD8+ T cells, elderly, brain cognition, HLA class I, healthy aging, CD4+ IFNγ+

## Abstract

There is increasing evidence that in humans the adaptive immunological system can influence cognitive functions of the brain. We have undertaken a comprehensive immunological analysis of lymphocyte and monocyte populations as well as of HLA molecules expression in a cohort of elderly volunteers (age range, 64–101) differing in their cognitive status. Hereby, we report on the identification of a novel signature in cognitively impaired elderly characterized by: (1) elevated percentages of CD8+ T effector-memory cells expressing high levels of the CD45RA phosphate receptor (Temra ^hi^); (2) high percentages of CD8+ T cells expressing high levels of the CD8β chain (CD8β^hi^); (3) augmented production of IFNγ by *in vitro* activated CD4+ T cells. Noteworthy, CD3+CD8+ Temra ^hi^ and CD3+CD8β^hi^ cells were associated with impaired cognition. Cytomegalovirus seroprevalence showed that all volunteers studied but one were CMV positive. Finally, we show that some of these phenotypic and functional features are associated with an increased frequency of the HLA-B8 serotype, which belongs to the ancestral haplotype HLA-A1, Cw7, B8, DR3, DQ2, among cognitively impaired volunteers. To our knowledge, this is the first proof in humans linking the amount of cell surface CD45RA and CD8β chain expressed by CD8+ Temra cells, and the amount of IFNγ produced by *in vitro* activated CD4+ T cells, with impaired cognitive function in the elderly.

## Introduction

A possible role for the immunological system in maintaining central nervous system (CNS) homeostasis has long been a matter of debate. Generally, it has been considered harmful in the context of neurodegenerative disorders with an autoimmune etiology (1). However, the accumulated evidence from clinical and experimental studies has consolidated the view that the innate and adaptive components of the immunological system are crucial players in neuronal homeostasis and cognitive function by fine-tuning the balance between neuroprotection and neurodegeneration (2–4). Thus, early studies in mice showed that CD4+ and CD8+ T cell deficiency was associated with cognitive dysfunction and deficient remyelination after spinal-cord injury, and that thymus-derived CD4+CD25+ Treg cells specific for myelin self-antigens were necessary to confer protection after injury to the CNS (5–7). Although similar studies in humans are lacking, there is recent evidence suggesting that innate and adaptive immunological cells modulate hippocampal neurogenesis and behavior through not-well understood mechanisms (8). In this respect, recent reports have shown that the human brain is inhabited by a large fraction of effector-memory CD8+ T cells expressing or not the CD45RA isoform, with some becoming tissue-resident upon expression of CD69 and CD103 (9). Resident CD8+CD69+ T cells in the human brain show increased expression of tissue homing and inhibitory receptors, and are low producers of granzymes (9). Some of these brain-resident CD8+ T cell features are shared by certain populations of peripheral blood effector-memory CD8+ T cells (10, 11), and are in accordance with experimental evidence suggesting that peripheral blood circulating CD8+ Temra cells migrate to the brain parenchyma (12, 13). In this respect, a number of clinical and experimental studies have shown that migration of peripheral blood effector-memory CD4+ and CD8+ T cells into the CNS may occur *via* the blood-cerebrospinal fluid barrier (BCSFB) and *via* the blood-brain barrier (BBB) (12–14) and that brain-resident T cells may leave and reenter the blood circulation (15). In this regard, a recent seminal study in Alzheimer’s disease patients has identified an immune signature that consists of expansions of CD8+ Temra cells in peripheral blood as well as in the cerebrospinal fluid produced by the choroid plexus (16).

In humans, the concept of cognitive function is closely linked with a successful aging process and both have been associated with expansions of NK-like CD8+ T cells, a heterogeneous pool of CD8+ T cells that include CD8+ Temra cells (17–19). A fraction of the expanded CD8+ Temra cells found in peripheral blood of aged healthy people, including centenarians, are claimed to be driven by human cytomegalovirus, a wide-spread virus found in young and aged people (20, 21). Yet, the CD8+ Temra cell expansions seen in peripheral blood may be caused by other factors, such as aging itself (18, 22), anomalies in signaling molecules (23, 24), inflammatory environments (25), physical exercise (26), and homeostatic cytokines (19, 27). Moreover, exceptional aging is not necessarily associated with high levels of disability (28), bringing up the question of which factors, endogenous and/or exogenous, may contribute to better cognitive function in the elderly (17). In this respect, recent human studies have provided evidence that expression of certain NK receptors (CD56 and NKG2D), but not others (NKG2A and KIR2DL1), by human NK-like CD8+ T cells is associated with better cognitive and physical function among elderly people (18). In line with these results, Serra-Miranda et al., described an immunological signature associated with better cognitive performance in healthy senior people that is characterized by low numbers of effector-memory CD4+ T cells and high numbers of B cells in peripheral blood (29). Although the molecular mechanisms used by the CD8+ T cells, and their receptors, to modulate cognitive, behavioral and physical functions in the elderly are presently uncertain, the involvement of secreted cytokines and other factors in response to the sensing of the inhabited environment are likely candidates in mediating this important biological function (19, 30, 31).

To further explore an association between immunological cells and cognitive function in humans, we undertook a comprehensive analysis of peripheral blood lymphocyte and monocyte populations in a cohort of elderly volunteers. The results revealed that the expression of CD45RA and CD8β by CD8+ T cells, the production of IFNγ by activated CD4+ T cells, and the presence of the HLA-B8 molecule constitute a novel immunological signature that discriminates between cognitively impaired and unimpaired elderly.

## Material and Methods

### Subjects and Classification Criteria

A total of 86 volunteers were recruited from elderly people from retirement homes and day care centers of the Beira Interior region of Portugal (EBIcohort, https://icon-cics.weebly.com/ebicohort-english.html). Blood from each volunteer was collected in EDTA tubes (10mL, for phenotype studies) and heparin tubes (4mL, for IFNγ studies) and assigned a double identification code, according with the Ethics Committee approved proposal. Coded samples were processed within hours of collection to obtain peripheral blood mononuclear cells (PBMC) and plasma. One mL aliquots of whole blood and plasma were cryopreserved in a −80°C freezer for later studies. PBMC were immediately phenotyped and functionally characterized.

Volunteers were evaluated by a trained team which assessed the volunteers using the Global Deterioration Scale (GDS), a revised Addenbrooke’s Cognitive Examination (ACE-R) test (32) and also performed physical activity analysis. For physical activity, a three-meter walking test was performed following previous described guidelines (33). The gait speed was determined as the distance traversed (3-meters) by the time between the first and the last step. The volunteers were classified in groups using the GDS, in which cognitive status is defined by GDS, ACE-R and clinical information. The groups are the following: A) Volunteers with no cognitive impairment (NCI) were those with GDS stages 1 and 2, having an ACE-R value indicative of normal cognitive impairment (106.49 ± 3.05% of minimal normal score) and did not have clinical indications of disease involving cognitive impairment; B) volunteers with mild cognitive impairment (MCI) were those with GDS stage 3, and having an ACE-R score slightly lower than minimal considered as normal (media of 73.6 ± 3.61% of minimal normal cognitive impairment level); C) volunteers with moderate to severe cognitive impairment (MtSCI, thereafter CI) were those with GDS stages 4 or above (34, 35), that include 14 volunteers that performed ACE-R tests (47.51 ± 4.91% of minimal normal cognitive impairment level), and 8 volunteers with clinical information indicating existence of dementia or Alzheimer disease, for which there was not possible to apply ACE-R test. All obtained data from volunteers (personal, clinical and evaluation data) were stored according with data protection regulation and legal directives.

Of the total cohort, a total of 34 volunteers were excluded from analysis. The exclusion criteria included withdrawal from the study, infection other than CMV, diagnosis of previous stroke, Parkinson’s disease, neoplasia, psychiatric disorders such as epilepsy, trauma or absence of clinical data. The remaining 50 volunteers included 13 volunteers with no cognitive impairment (NCI, 8 males and 5 females), 15 volunteers with mild cognitive impairment (MCI, 3 males and 12 females) and 22 volunteers with cognitive impairment (CI, 4 males and 18 females). For HLA typing two additional volunteers were studied, one male NCI and one male CI. This study was approved by the local Ethics Committee in accordance with the Declaration of Helsinki (Ref. Number CE-UBI-Pj-2017-012). All the participants or their legal representative gave their written informed consent.

### Cells and Flow Cytometry Studies

Peripheral Blood Mononuclear Cells (PBMC) were obtained from a cohort of elderly volunteers differing in their cognitive status after centrifugation over Lymphoprep (STEMCELL Technologies). Contaminating red blood cells (RBC) were lysed in RBC lysis solution (10 mM TRIS, 155 mM NH_4_Cl, pH 7.4) for 10 min at 37°C. For cell surface staining of lymphocytes, approximately 0.5x10^6^ PBMC were incubated in 96-well round-bottom plates at 4°C in the dark for 45 min with combinations of the different fluorochrome-conjugated antibodies, previously diluted in staining solution (Phosphate-Buffered Saline (PBS), 0.2% BSA, and 0.1% NaN_3_). For monocyte labeling, cells were first incubated with Human TruStain FcX (BioLegend) for 10 min at room temperature prior to cell surface staining. Appropriate combinations of fluorochrome-conjugated monoclonal antibodies against CD3, CD4, CD8α, CD8β, CD14, CD16, CD19, CD28, CD45RA, CD56, CD202b, CCR7, NKG2D, KIR2DL1, and IFNγ, together with irrelevant isotypes were used (**Supplemental Table 1**). After staining, cells were washed, acquired in a BD Accuri C6 (BD Biosciences) and analyzed using BD Accuri C6 software (BD Biosciences) or FlowJo software (FlowJo, LLC, for GMFI calculations). For lymphocytes, a minimum of 10,000 and a maximum of 20,000 events were acquired within the CD3+CD4+ and CD3+CD8+ T cell regions after gating on the lymphocyte region as determined by FSC and SSC. For monocytes, 20,000 events were acquired on the monocyte region as determined by FSC and SSC.

### Cell Activation and IFNγ Production

For IFNγ detection by activated T cells, 100μL of heparinized blood per test were diluted with 400μL of RPMI medium (Merck Millipore) and placed in 24-well plates. The diluted whole blood was stimulated by adding Cell Activation Cocktail containing PMA, Ionomycin and Briefeldin A (BioLegend) for 4 h in an incubator at 37°C and 5% CO_2_. After stimulation, cells were harvested and RBC were lysed twice in RBC lysis solution for 10 min at 37°C. Cells were then labeled with fluorochrome-conjugated monoclonal antibodies against cell surface receptors CD3, CD4, CD8 and CD28 in staining buffer in 96-well round-bottom plates for 45 min at 4°C in the dark. After extracellular labelling, cells were fixed for 30 min and permeabilized using eBioscience™ Intracellular Fixation & Permeabilization Buffer Set. After washing, cells were stained with FITC-conjugated anti-IFNγ or mouse IgG1-FITC (BioLegend, **Supplemental Table 1**) for 30 min at room temperature. After the washing steps, cells were resuspended in PBS, and whenever possible between 10-20 thousand events within the CD3+CD4+ and CD3+CD8+ T cell regions acquired using BD Accuri C6 flow cytometer (BD Biosciences) and analyzed using BD Accuri C6 software (BD Biosciences).

### Cytomegalovirus Seropositivity

For CMV detection, cryopreserved plasma samples were thawed and anti-CMV IgG antibodies detected by using 96-well micro-plate ELISA kits (Demeditec), according to manufacturer instructions. Tests were performed in duplicate and the amount of CMV-specific IgG antibody bound calculated using a BioRad xMark™ Microplate Absorbance Spectrophotometer. The concentration of IgG antibodies was calculated by comparing to a reference curve obtained with calibrators (ie, human serum diluted with PBS, with 1, 10, 30, 90 U/mL of anti-CMV IgG antibodies) following manufacturer instructions.

### HLA Determination

DNA was extracted from peripheral blood using the MagAttract^®^ DNA Blood Midi M48 kit or QIAamp DNA Stool Mini Kit (QS). HLA typing was accomplished using One Lambda^®^ LABTypeSSO kits at low resolution level (serology equivalent) for HLA-A, -B, -C, -DRB1, -DQA1 and -DQB1 loci followed by Luminex^®^ xMAP^®^ technology. Data were deduced in Fusion v4.2 software and are presented at serological equivalent or at antigen allele level when there is no serological equivalent.

### Statistical Analysis

Statistical analysis was performed using SPSS software (version 26, IBM) and statistical significance was defined as p<0.05. Graphs were done using GraphPad Prism 7 software. Continuous variables were expressed as the mean ± standard error of the mean (SEM). Differences in means among the three cognitive status’ groups were analyzed using one-way analysis of variance (ANOVA). Two-way ANOVA was used to examine the influence of two different categorical independent variables on one continuous dependent variable. When ANOVA showed significant differences, pairwise comparisons between means were tested using Post-Hoc Bonferroni multiple comparisons test. Comparison between the percentage of CD8+IFNγ T and CD4+IFNγ+ T cells and the percentage of CD28− cells among CD8β^lo^ and CD8β^hi^ T cells was assessed using paired samples T-test. Fisher’s Exact test was used to evaluate differences in the frequencies of categorical variables (eg., HLA antigens/serotypes, gender and CMV seropositivity) of unrelated samples (cognitive status’ groups). Z-test was used to evaluate differences within cognitive status’ groups, and adjusted p-values were calculated using Bonferroni method. Spearman’s ρ correlation coefficient was used to analyze the correlation between cognitive status’ groups and continuous variables. Pearson correlation was used to analyze the correlation between two continuous variables. MetaboAnalyst (version 4.0) was used for cluster analysis and data visualization (36). Heatmap was created using Euclidean distance measure, Ward clustering algorithm, normalized data, and showing only group averages.

## Results

### Main Peripheral Blood Mononuclear Cell Populations Among Elderly Differing in Their Cognitive Status

**Figure 1:**
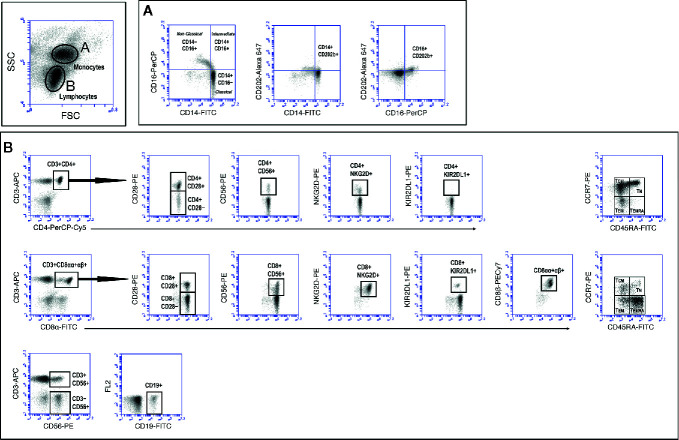
Gating strategy to study monocyte and lymphocyte populations. Peripheral blood mononuclear cells (PBMC) were isolated and stained as described in *Material and Methods* section. Labeled cells were acquired in a BD Accuri C6 flow cytometer and monocytes and lymphocytes discriminated according to FSC and SSC characteristics (top dot-plot). Markers were analyzed after creating and electronic gate around monocytes **(A)** and lymphocytes **(B)**. The different T cell markers expressed (y axes) were analyzed after creating electronic gates in CD3+CD4+ T cells (**B**, upper row) and CD3+CD8+ T cells (**B**, middle row). NK (CD3−CD56+), NKT (CD3+CD56+) and B (CD19+) cells were calculated as indicated (**B**, lower row).

Relevant clinical data of the different volunteers studied are shown in **Table 1**. The groups were age-matched, but the number of males in the NCI group (8 out of 13, 61%) was overrepresented in relation to the CI group (4 out of 22, 18%). However, two-way analysis of variance (ANOVA) to examine the influence of gender and cognition on the results obtained, showed that the results obtained are influenced by cognitive impairment (p<0.05) but not by gender. On the contrary, physical activity (as measured by three-meters walking) was statistically significantly different among groups (One-way Anova with Bonferroni’s correction, p=0.003, **Table 1**). Also shown in **Table 1** are the results of CMV seropositivity, which showed that 49 out of the 50 volunteers were IgG seropositive for CMV, as determined by ELISA. A thorough characterization of the different mononuclear cell populations present in peripheral blood samples was performed following the gating strategy illustrated in **Figure 1**. This strategy allowed us to characterize the three main populations of monocytes according to the expression of CD14 and CD16 into classical, intermediate and non-classical monocytes. Likewise, this strategy permitted to characterize T, B and NK populations as well as T cell subpopulations, according to the presence or absence of naive and differentiation markers, such as CD28, CCR7, CD45RA, CD56, NKG2D, and KIR2DL1. In addition, we extended further our analysis by including antibodies against the CD8β chain, besides the widely used against the CD8α chain. While antibodies against CD8α identify both CD8+ T cells and CD8+ NK cells, antibodies against CD8β exclusively detect CD8+ T cells. The results showed that elderly people differing in their cognitive status have no significant differences in the percentage of the different monocyte populations present in peripheral blood nor in the percentages of CD3+ T cells, CD4+ T cells, CD8+ T cells, CD19+ B cells, CD3−CD56+ NK cells, and CD3+CD56+ NKT cells (**Supplemental Figure 1**). We could also not detect any other statistically significant difference in the percentage of naive (Tn), central-memory (Tcm), effector-memory (Tem) and effector-memory CD45RA+ (Temra) in CD4+ and CD8+ T cells among the three groups. Likewise, we found no differences in the expression of CD28, CD56, NKG2D and KIR2DL1 among CD4+ and CD8+ T cells (see **Supplemental Figure 1**).

**Table 1:** Relevant data of the volunteers under study.

Clinical data				*P*-value^g^ ^/^ ^h^
Cognitive Status (number)^a^	NCI (n=13)	MCI (n=15)	CI (n=22)	–
Age, Mean ± SEM (range)	83.7 ± 2.4 (69-96)	82.5 ± 2.1 (64-96)	84.7 ± 1.9 (67-101)	NS^g^
Gender (M/F)	8/5	3/12	4/18	0.023^h^*
Gait Speed (m/s)	0.60 ± 0.05	0.41 ± 0.06	0.33 ± 0.04	0.003^g^**
Body Mass Index (BMI)	25.4 ± 2.6	23.1 ± 2.7	20.9 ± 3.4	NS^g^
CMV Seropositivy	12/13	15/15	22/22	NS^h^
**Lymphocytes (Mean ± SEM)**				***P*-value^g^**
% CD19+ (B cells)^b^	4.6 ± 0.8	5.1 ± 1.0	3.7 ± 0.6	NS
% CD3−CD56+ (NK cells)^b^	24.5 ± 3.4	25.0 ± 3.0	25.4 ± 3.2	NS
% CD3+CD56+ (NKT cells)^b^	8.1 ± 1.5	8.3 ± 1.5	5.3 ± 0.9	NS
% CD3+ T cells (T cells)^b^	66.6 ± 2.9	67.0 ± 2.4	67.9 ± 3.3	NS
% CD3^+^CD4^+^ T cells^b^	34.8 ± 4.1	40.3 ± 3.6	39.6 ± 2.3	NS
% CD4^+^ Tn ^c^	40.9 ± 6.5	34.6 ± 6.3	48.8 ± 3.4	NS
% CD4^+^ Tcm ^c^	31.6 ± 3.2	37.7 ± 4.2	28.7 ± 2.5	NS
% CD4^+^ Tem ^c^	20.1 ± 4.8	23.4 ± 3.7	16.6 ± 2.1	NS
% CD4^+^ Temra ^c^	7.3 ± 2.6	4.4 ± 1.4	5.9 ± 1.7	NS
% CD3^+^CD8^+^ T cells^b^	31.4 ± 4.9	26.0 ± 2.9	27.7 ± 2.5	NS
% CD8^+^ Tn ^d^	11.9 ± 2.5	14.3 ± 2.6	14.3 ± 1.9	NS
% CD8^+^ Tcm ^d^	6.8 ± 1.2	9.2 ± 2.0	7.5 ± 1.3	NS
% CD8^+^ Tem ^d^	19.5 ± 4.1	20.2 ± 3.7	17.4 ± 3.0	NS
% CD8^+^ Temra ^d^	61.8 ± 5.1	56.4 ± 4.5	60.8 ± 4.3	NS
**Monocytes (Mean ± SEM)**				
% CD14+CD16− (classical)^e^	85.5 ± 1.8	82.1 ± 3.6	81.4 ± 2.4	NS
% CD14+CD16+ (intermediate)^e^	4.4 ± 0.7	5.8 ± 0.9	6.8 ± 1.2	NS
% CD14−CD16+ (non-classical)^e^	6.0 ± 0.9	7.8 ± 2.9	7.0 ± 1.4	NS
% CD14+CD202b+^e^	3.5 ± 0.9	3.1 ± 0.4	4.7 ± 1.0	NS
% CD16+CD202b+^e^	3.0 ± 0.7	2.2 ± 0.5	3.5 ± 0.9	NS
**HLA Serological Antigens (%)** ^f^	NCI (n=14)	MCI (n=15)	CI (n=23)	***P*-value^h^**
HLA-A03	14.3	23.3	4.3	0.044***
HLA-B08	0.0	6.7	17.4	0.034*
HLA-C12	17.9	26.7	6.5	0.045***
HLA-DQB1_6	14.3	26.7	6.5	0.045***

a Cognitive status determined by Global Scale Deterioration (see Materials and Methods section). NCI, No Cognitive Impairment; MCI, Mild Cognitive Impairment; CI, Cognitive Impairment.

b Percentages determined after gating in the lymphocyte gate.

c Percentages determined after gating in CD3+CD4+ T cells.

d Percentages determined after gating in CD3+CD8+ T cells.

e Percentages determined after gating in the monocyte gate.

f Percentages determined after applying the following formula: # of seropositive antigens ×100 /(# of volunteers per group x 2). In the serological analysis two additional volunteers (one NCI and one CI) without flow cytometry data were included.

g P-values determined by One-way ANOVA test; NS, Not significant.

h P-values determined by Fisher’s Exact test with Post-Hoc z-test to compare column proportions with p-values adjusted using Bonferroni’s method.

*Statistically significantly different between NCI and CI.

**Statistically significantly different between NCI vs. MCI (p=0.045) and NCI vs. CI (p=0.003).

***Statistically significant different between MCI and CI.

### The Level of Expression of CD45RA and CD8β in CD8+ T Cells Discriminates Between Cognitively Unimpaired and Cognitively Impaired Elderly

**Figure 2:**
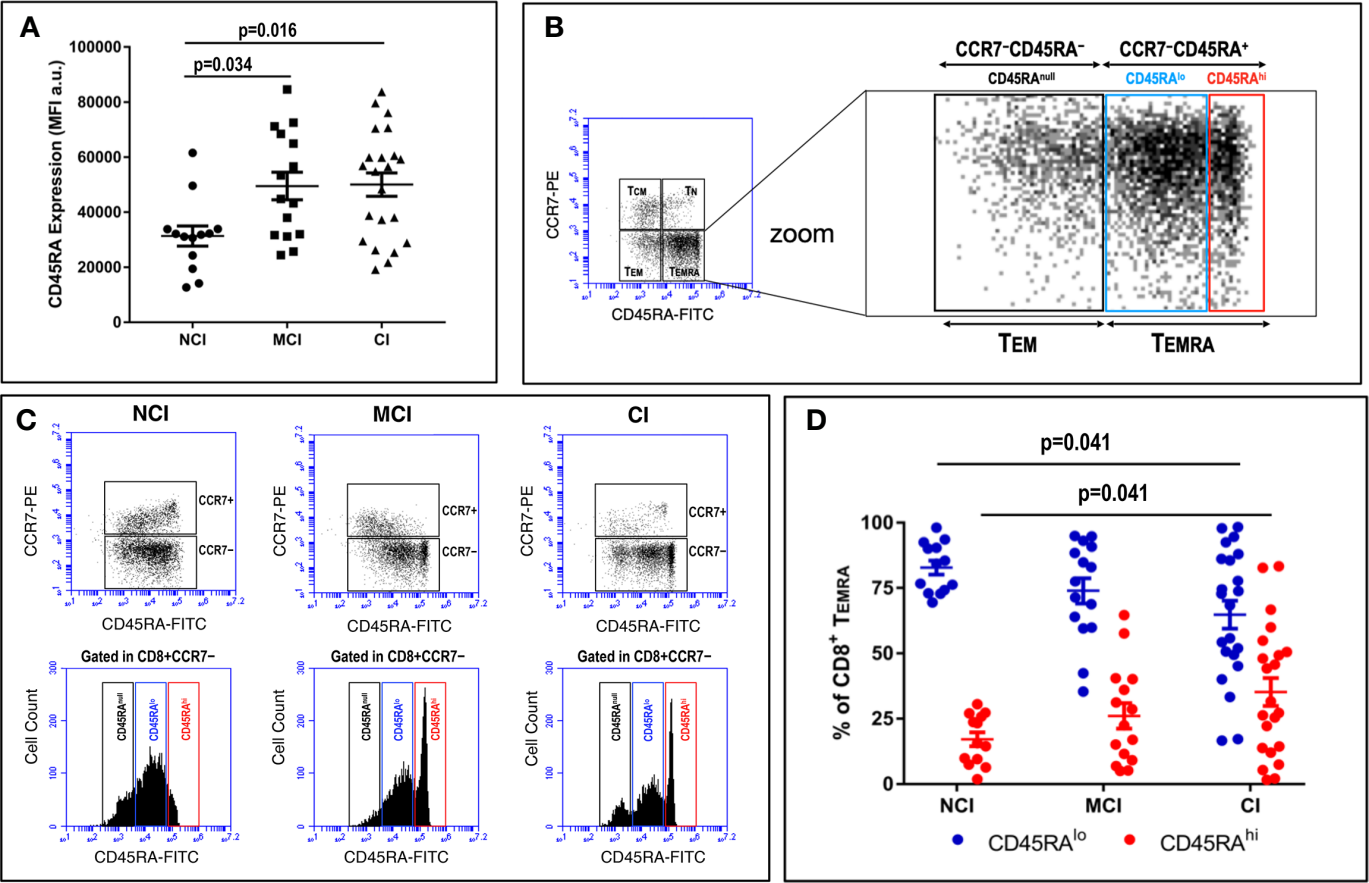
PBMC were isolated, stained and acquired as described in the legend of **Figure 1**. **(A)** Mean Fluorescence Intensity (MFI) values (a.u., mean ± SEM) of the expression of CD45RA after gating on CD3+CD8+CD45RA+ T cells in the three groups of elderly volunteers. Statistically significant differences between groups are indicated (ANOVA with Bonferroni’s correction). (**B**, left) Dot-plot of CCR7 vs. CD45RA expression in CD3+CD8+ gated T cells showing naive (Tn), central-memory (Tcm), effector-memory (Tem) and effector-memory CD45RA+ (Temra) cells. (**B**, right) Zoom of dot-plot lower quadrants (Tem + Temra) showing the existence of distinct subpopulations according to the level of expression of CD45RA: CD45RA^null^, CD45RA^lo^, and CD45RA^hi^. **(C)** Plots of CCR7 vs. CD45RA expression in CD3+CD8+ gated T cells (upper row dot-plots) and CD45 expression (lower row histograms) in three representative volunteers. Histograms show CD45RA expression in CD3+CD8+CCR7− gated Tem+Temra cells. Three distinct CD8+CD45RA T cell populations differing in their MFI values can be distinguished: CD8+CD45RA^null^, CD8+CD45RA^lo^ and CD8+CD45RA^hi^. **(D)** Graph showing the relative percentage of CD45RA^lo^ (blue circles) and CD45RA^hi^ (red circles) in CD8+CCR7−CD45RA+ gated T cells in the three volunteer groups (mean ± SEM). Statistically significant differences between groups are indicated (ANOVA with Bonferroni’s correction). a.u., arbitrary units.

Even though we could not detect any significant difference in the percentages of Temra cells among the three volunteer groups, a thorough analysis of the level of expression of CD45RA among CD8+ Temra cells revealed differences in the amount of CD45RA. **Figure 2A** shows that expression of CD45RA at the cell surface of CCR7−CD8+ Temra cells, as determined by mean fluorescence intensity (MFI) values, was statistically significantly lower in no-cognitively impaired (NCI) volunteers when compared to both mild cognitively impaired (MCI) volunteers (31314 ± 3667 vs. 49489 ± 4996, mean ± SEM, p=0.034) and cognitively impaired (CI) volunteers (31314 ± 3667 vs. 50017 ± 4233, mean ± SEM, p=0.016). In order to avoid the influence of outliers in these results, we also compared geometrical mean fluorescence intensity values (GMFI). The results of this analysis were identical to the results of MFI. Thus, there were statistically significant differences between the 3 groups (One-way ANOVA, p= 0.008), with the CI group presenting higher GMFI values. Moreover, after application of the Bonferroni test for multiple comparisons, statistically significant differences were found between the NCI and MCI groups (p=0.029), and between NCI and CI groups (p=0.011). This finding prompted us to perform an in-deep analysis of the CD8+ Temra population. The results showed the presence of two distinct CD45RA subpopulations (**Figure 2B**). One population expressing high levels of CD45RA (henceforth designated as CD8+ Temra ^hi^) and another population expressing lower levels of CD45RA (henceforth designated as CD8+ Temra ^lo^). When analyzed individually, this pattern of expression revealed marked differences between the NCI volunteers and the MCI and CI volunteers, with the two later showing a noticeable and sharp CD8+ Temra ^hi^ population (**Figure 2C**). Determination of the relative percentage of CD8+ Temra ^hi^ and CD8+ Temra ^lo^ cells within CD8+ Temra revealed marked differences between the NCI and CI groups (**Figure 2D**). Thus, in CI volunteers the relative percentage of CD8+ Temra ^hi^ cells was statistically significantly increased by two-fold when compared to NCI volunteers (35.2 ± 5.3 vs. 17.1 ± 2.7, mean ± SEM, p=0.041). Accordingly, the relative percentage of CD8+ Temra ^lo^ cells was markedly reduced (64.8 ± 5.3 vs. 82.9 ± 2.7, mean ± SEM, p=0.041). Similar results were observed when the percentage of CD8+ Temra ^hi^ and CD8+ Temra ^lo^ cells within CD8+ T cells were compared (data not shown). Importantly, cross-correlation studies revealed that the relative percentage of CD8+ Temra ^hi^ and CD8+ Temra ^lo^ cells were positively (ρ=0.289, p=0.042) and negatively (ρ=−0.289; p=0.042), respectively, associated with cognitive status.

**Figure 3:**
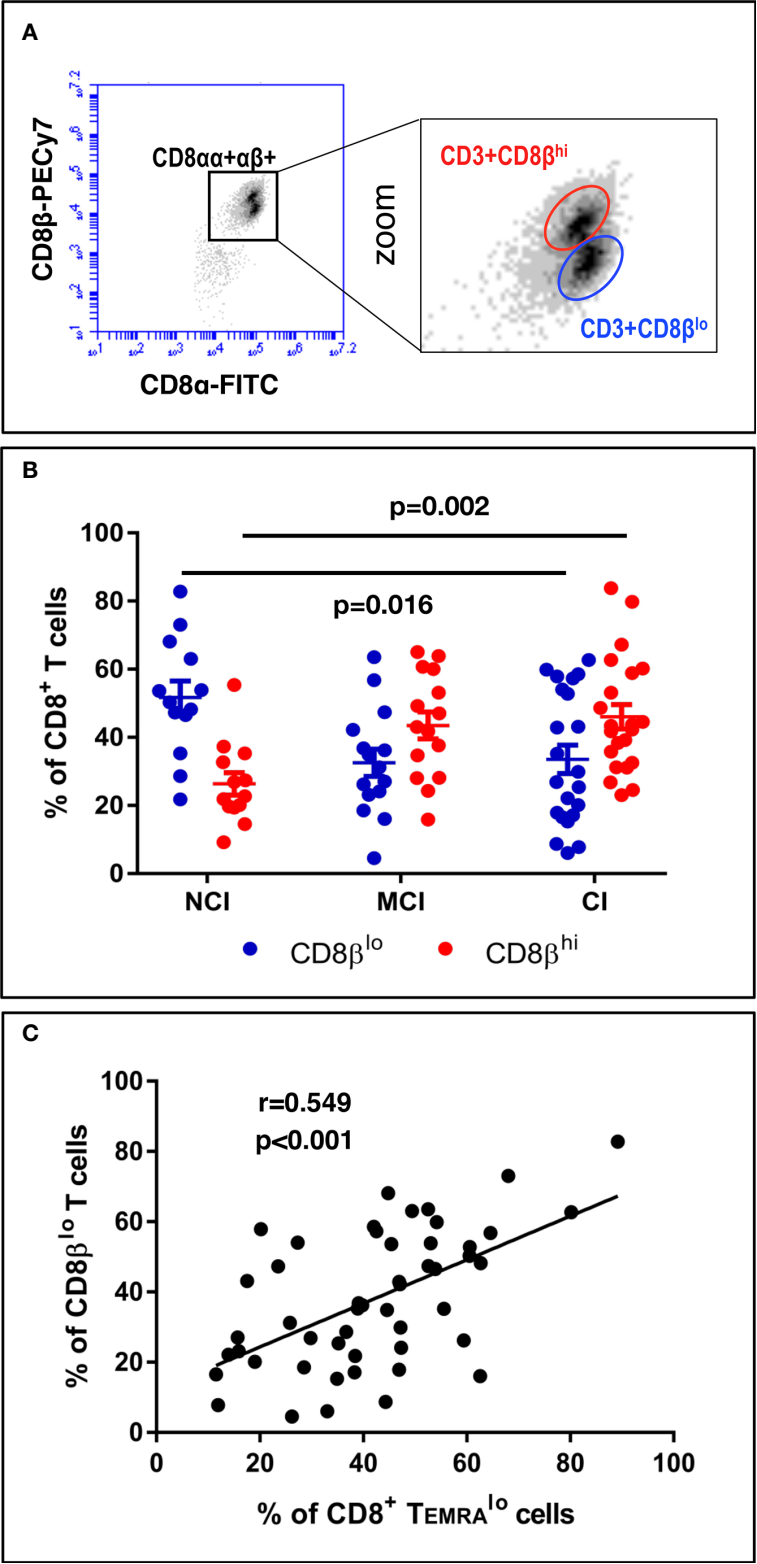
PBMC were isolated, stained and acquired as described in the legend of **Figure 1**. (**A**, left) Dot-plot of CD8β vs. CD8α expression in CD3+CD8+ gated T cells. (**A**, right) Zoom of CD3+CD8αβ+ T cells showing the existence of two distinct populations according to the level of expression of CD8β: CD3+CD8β^hi^ and CD3+CD8β^lo^. **(B)** Graph showing the percentage of CD3+CD8β^lo^ (blue circles) and CD3+CD8β^hi^ (red circles) in CD3+CD8+ gated T cells in the three volunteer groups (mean ± SEM). Statistically significant differences between groups are indicated (ANOVA with Bonferroni’s correction). **(C)** Scatter-plot showing a significant positive correlation between the percentages of CD8+ Temra ^lo^ and CD8β^lo^ T cells (Pearson correlation, n=50). a.u., arbitrary units.

On the other hand, the large majority of studies characterizing phenotypically and functionally CD8+ T cell populations in humans have used antibodies that recognize the CD8α chain. Here, we show that the use of antibodies against the CD8β chain revealed the existence of two distinct CD8+ T cell subpopulations based on the level of expression of the CD8β chain: CD3+CD8β^lo^ T cells and CD3+CD8β^hi^ T cells (**Figure 3A**). Determination of the percentages of these two CD8β+ T cell populations showed that NCI volunteers have higher percentages of CD3+CD8β^lo^ T cells than CI volunteers (51.7 ± 4.8 vs. 33.5 ± 4.1, mean ± SEM, p=0.016), while presenting lower percentages of CD3+CD8β^hi^ T cells (26.3 ± 3.3 vs. 46.0 ± 3.6, mean ± SEM, p=0.002) (**Figure 3B**). Given the resemblances between the expression of CD45RA and CD8β in cognitively unimpaired and cognitively impaired elderly, we decided to ascertain whether they were related populations. The existence of a robust positive correlation between CD8+ Temra ^lo^ cells and CD8β^lo^ T cells (**Figure 3C**, r=0.549, p<0.001) is a strong indication that they are similar CD8+ T cell populations. Finally, in order to better visualize the distribution of the aforementioned CD8+ T cell populations among the different cognitive groups, a heatmap representation was generated. This analysis revealed the existence of two clusters whereby the NCI group was enriched for CD8+ Temra ^lo^ and CD8β^lo^ T cells, while the CI group was enriched for CD8+ Temra ^hi^ and CD8β^hi^ T cells (**Figure 5**).

Comparison of the expression of CD28 by CD8β^lo^ and CD8β^hi ^T cells showed that the former are more enriched for CD28− cells (74.6 ± 2.3 vs. 45.5 ± 3.7, mean ± SEM, p<0.001). Interestingly, cross-correlation analysis revealed that the percentage of CCR7−CD45RA^lo^, but not of CCR7−CD45RA^hi^ CD8+ T cells, positively correlated with the percentage of CD8+CD28− T cells (r=0.506, p<0.001). These results indicate that CD8+CD45RA^lo^ and CD3+CD8β^lo^ T cells are predominantly CD28−, which is in accord with previous studies (37).

### Activated CD4+ T Cells From Cognitively Impaired Volunteers Produce Higher Levels of IFNγ Than Cognitively Unimpaired Volunteers

**Figure 4:**
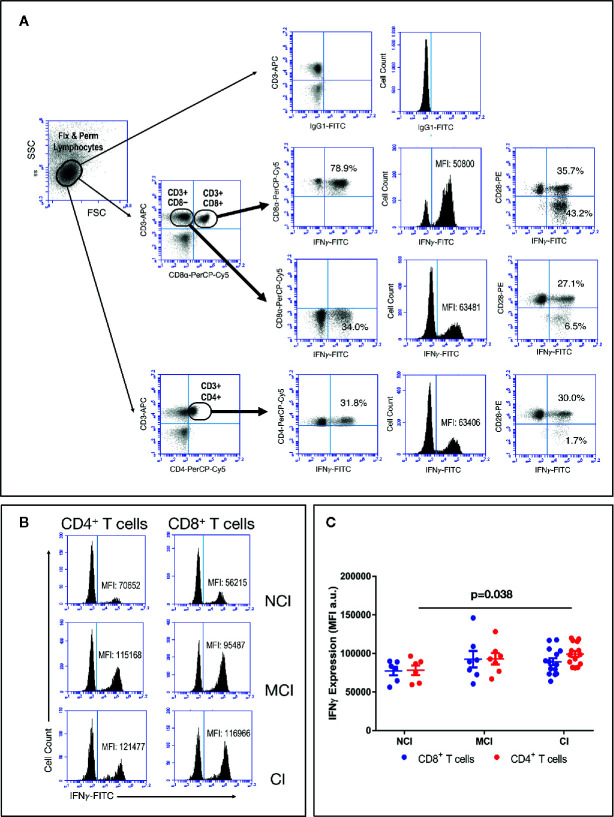
PBMC were isolated, activated with PMA+Iono for 4 h, washed and stained for CD3, CD4, CD8 and CD28, then fixed & permeabilized and stained again for IFNγ, and acquired in an Accuri C6 flow cytometer, as described in the *Material and Methods* section. **(A)** Graph illustrating the gating strategy to analyze IFNγ expression by activated T cells. Results show a representative experiment of the percentage of CD4+ and CD8+ T cells expressing intracellular IFNγ Upper graphs show background staining using an irrelevant IgG isotype. Percentages of CD3+CD4+IFNγ+ T cells and CD3+CD8+IFNγ+ T cells, as well as IFNγ MFI values are shown. **(B)** Illustrative results of IFNγ MFI values in activated CD4+ (left histograms) and CD8+ (right histograms) T cells from representative volunteers. **(C)** Graph showing MFI values of intracellular IFNγ (mean ± SEM) in gated CD4+ (red circles) and CD8+ (blue circles) T cells. Statistically significant differences between groups are indicated (ANOVA with Bonferroni’s correction).

**Figure 5:**
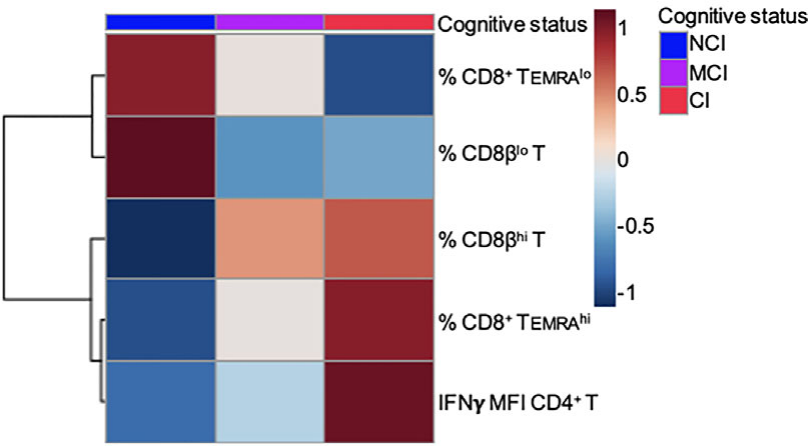
Heatmap showing two clusters of variables, Cluster 1 (% CD8+ Temra ^lo^ and % CD8β^lo^) and Cluster 2 (% CD8+ Temra ^hi,^ % CD8β^hi^ and IFNγ MFI CD4+ T), associated with no cognitively impaired (NCI) and cognitively impaired (CI) volunteers, respectively.

In order to evaluate production of IFNγ by activated T cells, a measure of the effector/regulatory potential of T cells, among the elderly groups, we stimulated PBMC with a combination of PMA and Ionomycin (Iono), followed by flow cytometry studies using combinations of anti-CD3, anti-CD28, anti-CD4, anti-CD8, and anti-IFNγ antibodies. Since PMA+Iono is known to induce a marked down-regulation of the CD4 receptor, we studied the production of IFNγ by CD4+ T cells by analyzing CD3+CD4+ T cells as well as CD3+CD8− T cells. As shown in **Figure 4A**, the percentage of CD4+IFNγ+ T cells (dot-plots) as well as the level of IFNγ produced by CD4+ T cells (histograms) were almost identical in either anlysis. Likewise, we observed that the percentage of CD8+IFNγ+ T cells in response to PMA+Iono was about two-fold higher than the percentage of CD4+IFNγ+ T cells (79.5 ± 2.6 vs. 35.4 ± 2.9, respectively, mean ± SEM, p<0.001). This two-fold increase in the percentage of IFNγ-producing CD8+ T cells was observed regardless of the cognitive status of the volunteers (data not shown). However, when we analyzed the actual level of expression of intracellular IFNγ by activated CD4+ and CD8+ T cells, by determining the mean fluorescence intensity (MFI) values, we observed higher levels of IFNγ in activated CD4+ T cells from CI volunteers when compared to the NCI volunteers, with the MCI group displaying intermediate values (**Figure 4B**). As a result, the levels of expression of intracellular IFNγ in activated CD4+ T cells observed in the CI group were statistically significantly higher than the levels of expression observed in the NCI group (99124 ± 3821 vs. 78069 ± 6236, mean ± SEM, p=0.038) (**Figure 4C**). Importantly, cross-correlation studies revealed that the values of MFI for IFNγ in activated CD4+ T cells correlated positively with cognition scores (ρ=0.439, p=0.022). In line with the heatmap representation generated for the CD8+ T cell populations, the CI group was enriched for CD4+ T cells producing high levels of IFNγ, contrasting with the NCI group, where the expression was lower (**Figure 5**).

### Higher Prevalence of the HLA-B8 Serotype in Cognitively Impaired Elderly

**Figure 6:**
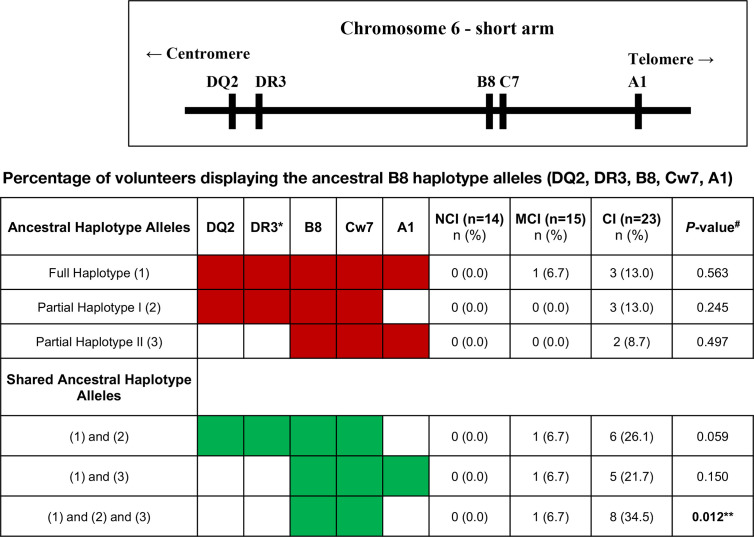
Upper figure represents part of the major histocompatibility complex (MHC) region, also known as the human leukocyte antigen (HLA) region on the short arm of chromosome 6. The table beneath shows the ancestral B8 haplotype alleles or its partial fragments (in red). The haplotypes were reconstructed by imputation of the allele markers and are putative, because it was not possible to strictly prove their segregation from family studies. The number of volunteers and the percentage within the cognitive status groups are shown. The shared B8 ancestral haplotypes are also shown (in green). *DR17 serotype; NCI, No-Cognitive Impairment; MCI, Mild Cognitive Impairment; CI, Cognitive Impairment. ^#^P-values were calculated using Fisher’s Exact Test with Post-Hoc z-test to compare column proportions with p-values adjusted using Bonferroni’s method. **Statistically significantly different between NCI and CI groups.

All the volunteers that were enrolled in this study were HLA typed for HLA-A, -B, -C, -DRB1, -DQA1 and -DQB1 loci and data presented at serological equivalent or at antigen allele level when there was no serological equivalent. The results of this analysis revealed that one-sixth of chromosomes of the CI volunteers expressed the HLA-B8 in heterozygosity, which contrasted with its complete absence in the NCI volunteers (17.4 vs. 0.0%, respectively, p=0.034). In other words, one-third of the CI volunteers were HLA-B8. Analysis of other HLA alleles revealed that a large percentage of the HLA-B8+ volunteers presented HLA alleles of the so-called ancestral haplotype, ie, HLA-DQ2, HLA-DR3, HLA-B8, HLA-Cw7, and HLA-A1. Further analysis revealed that that the HLA-B8 and HLA-Cw7 alleles were overrepresented among the CI group (**Figure 6**). No other significant differences were observed in the expression of other HLA serotypes between NCI and CI volunteers (**Figure 6**). The only finding was an increase in the frequency of MCI volunteers expressing the HLA-A3, HLA-C12 and HLA-DQB1_6 serotypes when compared to CI volunteers (see **Table 1**). Interestingly, analysis of the prevalence of HLA-B8 among the volunteers revealed that the percentage of CD3+CD8β^hi^ T cells, but not the percentage of CD8+CD45RA^hi^ T cells, is statistically significantly increased in volunteers displaying the HLA-B8 serotype (53.6 ± 5.2 vs. 37.2 ± 2.5, mean ± SEM, p=0.008).

## Discussion

During the last years there has been a change of dogma whereby T cells are also endowed with properties that promote behavioral improvement, brain plasticity and cognition (4, 38–41). Indeed, recent studies in clinical and experimental neurodegenerative settings have provided important insights into the neuroprotective role of T cells (42–44). Even though the molecular cues behind these associations are presently uncertain, these studies suggest that certain effector-memory T cell populations may favor a healthy aging, while others may be detrimental (18).

In order to gain further insights into the relationship between T cell populations in peripheral blood and healthy aging, we undertook a comprehensive immunological analysis of lymphocyte and monocyte populations as well as of HLA molecules expression in an aged-matched cohort of elderly volunteers differing in their cognitive status. Our results show for the first time that a subpopulation of CD3+CD8+ T cells characterized by the expression at the plasma membrane of high levels CD45RA (CD8+ Temra ^hi^) and high levels of the CD8β chain (CD3+CD8β^hi^) is overrepresented in elderly people with impaired cognitive status. On the contrary, elderly people with unimpaired cognitive status have CD3+CD8+ T cells characterized by the expression of low levels of cell surface CD45RA (CD8+ Temra ^lo^) and CD8β (CD3+CD8β^lo^). Likewise, we show that CD4+ T cells from elderly people with impaired brain cognitive status activated *in vitro* with PMA/Ionomycin produce about two-fold more IFNγ than elderly people with unimpaired brain cognitive status. Finally, we report that the HLA-B8 serotype is absent in the group of elderly people with unimpaired brain cognitive but present in 34% of elderly people with impaired brain cognitive status. More precisely one-sixth of chromosomes of the CI volunteers (17.4%) expressed the HLA-B8 in heterozygosity. On the one side, these results can be interpreted as meaning that CD8+ Temra ^hi^ and CD8β^hi^ T cells, together with high IFNγ-producing CD4+ T cells and the presence of HLA-B8, are immunological biomarkers associated with unhealthy aging (ie, impaired brain cognitive function). On the other side, the same results can be interpreted to mean that CD8+ Temra ^lo^ and CD8β^lo^ T cells are immunological biomarkers associated with healthy aging (ie, unimpaired brain cognitive function). In any case, these are novel and insightful data that point to selected subsets of highly differentiated CD8+ T cells as potential protectors of normal cognitive functions, as proposed by others (18, 40), and substantiate the double-edged role of IFNγ on cells of the CNS (45). Moreover, the finding of a prevalence of the HLA-B8 serotype among cognitively impaired volunteers unveils a novel association between impaired cognition and an HLA class I allele that is worthwhile confirming on a larger scale. The existence of two populations of circulating human CD3+CD8+ T cells differing in the expression of the tyrosine phosphatase isoform CD45RA and the CD8β chain is not new and has been previously reported (46). However, to our knowledge, this is the first proof in humans that the levels of expression of CD45RA and CD8β by CD8+ T cells are associated with cognition in a cohort of elderly volunteers.

These results are highly relevant because they suggest that CD8+ Temra ^lo^ and CD8β^lo^ T cells are related subsets that might play important roles in keeping brain’s cognitive function. Thus, they are highly differentiated oligoclonal CD8+ T cells that descend directly from expanded CD8αβ T cells *in vivo*, after down-regulation of the CD8β chain (37, 47) and re-expression of CD45RA (48, 49). Moreover, CD8αβ^lo^ T cells and CD8+ Temra ^lo^ cells are majoritarily CD28−, express CD45RA, respond poorly to TCR-mediated signals, and secrete IFNγ, perforin and granzyme upon stimulation (37, 47, 50, and this study). Interestingly, the CD8+ Temra ^lo^ cells described in this study resemble the CD8+ Temra+ cells described by Romero et al. (51) but using a different combination of surface markers. In this respect, a recent study has shown that the percentages of CD8+ Temra cells in peripheral blood and cerebrospinal fluid (CSF) of a human cohort encompassing mild cognitive impairment (MCI) and Alzheimer’s disease (AD) patients are negatively associated with cognition. The authors discuss the likelihood that the presence of CD8+ Temra cells in the CSF may promote neurodegeneration through their cytotoxic effector function (16). Our findings with a cohort of elderly people extend these results by showing that not all CD8+ Temra are necessarily negatively associated with cognition, but only those expressing high levels of CD45RA, ie, CD8+ Temra ^hi^ cells. At the same time, we have unveiled an association between CD8+ Temra ^lo^ cells and preserved brain cognitive functions. Therefore, while CD8+ Temra ^lo^ cells and CD8αβ^lo^ T cells might constitute potential pro-cognitive immunological biomarkers that favor brain’s cognitive function, CD8+ Temra ^hi^ cells and CD8β^hi^ T cells might constitute potential detrimental immunological biomarkers that disfavor brain’s cognitive function and healthy aging. Although previous studies have shown an association between high percentages of CD8+ Temra cells and infection by CMV (20, 21), the fact that 49 out of 50 elderly volunteers were CMV seropositive argues against the possibility that the differences found between NCI and CI volunteers is due to CMV infection. Nevertheless, the possibility that the CD8+ Temra ^hi^ cells found in the CI volunteers is being driven in some unidentified way by HCMV or other viral antigens cannot be discarded completely.

Even though the environmental signals driving the increase in the levels of CD45RA and the downmodulation of the CD8β chain by circulating effector-memory CD8+ T cells are uncertain, TCR-mediated stimulation and cytokines such as IL-1, IL-2 and IL-6, but not IFNγ, are known to regulate their expression (37, 52–57). Of note, IL-1/IL-6 are increased in neurodegenerative disorders such as Alzheimer’s and Parkinson’s disease, as well as in cognitive dysfunction (58, 59). Moreover, in animal models, IL-1 was shown to influence cognitive function by affecting long-term potentiation and possibly neurogenesis, while IL-6 impacts cognitive function *via* effects on neurogenesis and synaptic plasticity (60). These findings warrant further investigations to elucidate what signals enhance CD45RA expression by circulating CD8+ Temra ^lo^ cells and, at the same time, slow down CD8β chain downmodulation by CD8β^hi^ T cells during aging, and whether these changes are associated with the secretion of IL-1/IL-6 by CNS parenchymal cells. Regarding IFNγ, prior animal studies have shown that low doses can stimulate neurogenesis and induce protective signaling pathways in microglia, oligodendrocytes and primary neurons, thus raising the possibility of playing a role in development and repair of the CNS (61, 62). However, when present at high levels it induces disease worsening effects in both glial cell types and has detrimental effects on cognitive function, perhaps by inhibiting neural stem/progenitor cell proliferation (63, 64). These, apparently, contradictory biological effects of IFNγ are in accord with the opposing roles of this cytokine on cells of the CNS (45). In this respect, Gate et al. reported that production of IFNγ by activated CD8+ Temra cells was increased in the cohort of MCI and AD patients, suggesting that it may play a harmful role (16). In this scenario, the higher levels of IFNγ produced by CD4+ T cells upon activation found in the CI volunteers constitute a further detrimental immunological biomarker in the elderly. Although in our study, IFNγ production by activated CD8+ T cells was also increased in the CI group in comparison to the NCI group, this difference did not reach statistical significance.

Finally, the prevalence of the HLA-B8 serotype in volunteers with impaired cognition adds to the body of knowledge linking HLA-class I molecules with disease, a finding worthwhile confirming on a larger scale. If it proves true in a large cohort study, the role of HLA-B8 might give clues as where to go next in elucidating cognitive impairment in the elderly. In this regard, it is worth mentioning that HLA-B8 is part of the so-called “autoimmune” ancestral haplotype (HLA-DQ2, HLA-DR3, HLA-B8, HLA-Cw7, and HLA-A1) which is carried by most Caucasians and known to be positively associated with autoimmune disorders and by elevated circulating levels of inflammatory cytokines, such as IL-1 and TNFα (65). Thus, to our knowledge, this is the first report showing and association between cognitive impairment and the presence of HLA-B8, or alleles of the ancestral haplotype. Given the special features of HLA-class I molecules, we propose that the association found between the presence of alleles of the ancestral haplotype, namely HLA-B8 and HLA-Cw7, and cognitive impairment in the elderly cohort could result from three circumstances. First, as a result of the role of HLA-class I molecules as peptide presenting molecules that drive CD8+ T cell expansion and differentiation (66). In this regard, the study of Gate et al., is the first report showing that CD8+ T cells found in the CSF of AD patients carry a clonal TCR specific for an EBV-derived peptide presented by HLA-B8 (16). However, as pointed out by the authors, the EBV-specific clones detected in the study were not the most highly expanded ones and the data are not evidence of a causal link between EBV infection and AD (16). Second, as a result of the non-immunological functions of HLA-class I molecules (67), whereby these could modulate receptor-mediated signaling and endocytosis in CNS cells, and regulate brain function and plasticity (68, 69). In this context, it is worth mentioning recent studies in animal models showing that interactions between MHC-class I molecules and the insulin receptor in the brain could regulate neuronal insulin sensitivity in the aging and diseased brain (70). Third, as a result of shedding of cell surface HLA-class I molecules into the extracellular media, where they can exert immunoregulatory functions on neighboring cells (71). In this respect, a potential role for shed MHC-I molecules as modulators of neurodevelopment and neurorepair responses has been reported (72).

Although the possibility that common factors might impact simultaneously immune system and cognitive impairment cannot be disregarded, the results of this study reinforce the view that cells of the adaptive immunological system, namely highly differentiated CD8+ T cells, might play a pro-cognitive role in humans. On the other hand, the possibility that changes in cognition could also alter behavior/lifestyle and that this may then alter immune composition is an interesting possibility that deserves further investigation. Therefore, whether the CD8+ Temra ^hi^ cells, CD8β^hi^ T cells, and IFNγ-producing CD4+ T cells identified in this study as potential deleterious biomarkers of brain’s cognitive function in the elderly, are truly detrimental needs further investigations. If these adaptive T cell subsets are confirmed to have negative effects in brain’s cognitive function, they may be subjected to therapeutical manipulation. Similarly, if the CD8+ Temra ^lo^ and CD8β^lo^ T cells are corroborated as pro-cognitive, further investigations must address the mechanisms involved and how to guarantee their maintenance in peripheral blood.

## Data Availability Statement

The data that support the findings of this study are available on request from the corresponding author. The data are not publicly available due to privacy or ethical restrictions.

## Ethics Statement

This study was approved by the local Ethics Committee in accordance with the Declaration of Helsinki (Ref. Number CE-UBI-Pj-2017-012). All the participants or their legal representative gave their written informed consent.

## Author Contributions

FAA and EMC designed the experiments, analyzed data, and wrote the manuscript. AJE, DR-F, and SEA performed the experiments and analyzed data. AS, APA, and IV collected blood samples and clinical data. AM performed HLA typing. AJE, DR-F, ITO, and EMC performed statistical analysis. OL and AMF analyzed data. AJE contributed to write the manuscript. All authors contributed to the article and approved the submitted version.

## Funding 

This study was funded by FEDER funds through the POCI–COMPETE 2020—Program Competitiveness and Internationalization in Axis I—strengthening research, technological development, and innovation (Project No. 007491) and from National Funds by FCT—Foundation for Science and Technology (Project UID/Multi/00709/2020). ITO is a grantee of C4-UBI, Cloud Computing Competence Centre, University of Beira Interior. AJE, DR-F, SEA, AS, and APA were supported by fellowships from Project Nº. 007491.

## Conflict of Interest

The authors declare that the research was conducted in the absence of any commercial or financial relationships that could be construed as a potential conflict of interest.

## Supplementary Material

Supplementary Figure 1 T cell populations in elderly volunteers. PBMC were isolated, stained and acquired as described in the legend of **Figure 1**. The distribution of the different T cell populations (percentage, mean ± SEM) in the three volunteer groups (NCI, MCI, CI) is shown in the different graphs.

## Abbreviations

Temra, effector-memory CD45RA+ T cells; CMV, cytomegalovirus; GDS, global deterioration scale; NCI, no cognitive impairment; MCI, mild cognitive impairment; CI, cognitive impairment.
