# Supplementary figures and images for: CD45RA, CD8β, and IFNγ Are Potential Immune Biomarkers of Human Cognitive Function
Source: Front Immunol. 2020 Nov 25;11:592656. doi: 10.3389/fimmu.2020.592656 (PMC7723833; doi:10.3389/fimmu.2020.592656)

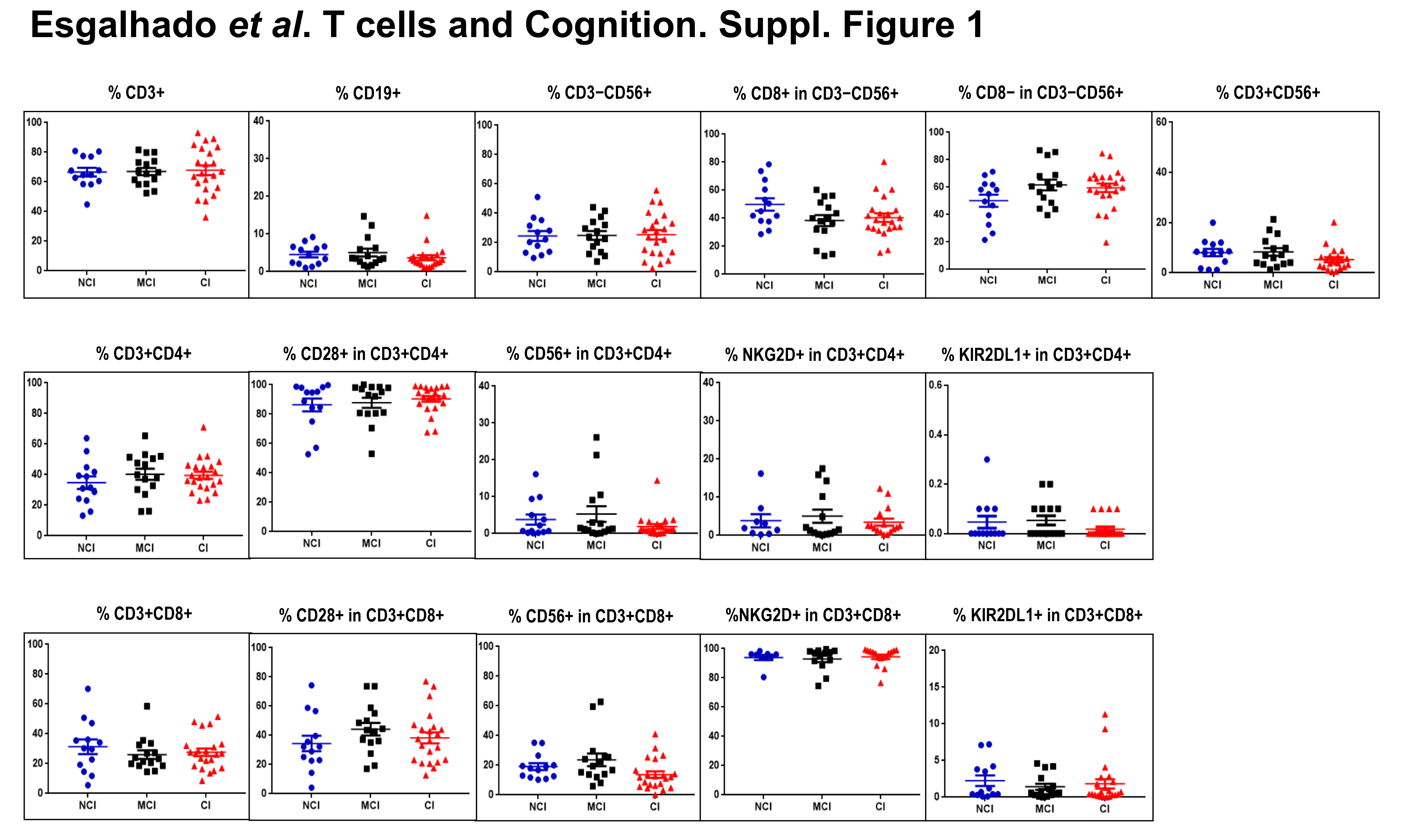

Supplement: Supplementary Figure 1 — T cell populations in elderly volunteers. PBMC were isolated, stained and acquired as described in the legend of Figure 1 . The distribution of the different T cell populations (percentage, mean ± SEM) in the three volunteer groups (NCI, MCI, CI) is shown in the different graphs. [file Image_1.tif]
