# Supplementary material for: CD45RA, CD8β, and IFNγ Are Potential Immune Biomarkers of Human Cognitive Function
Source: Front Immunol. 2020 Nov 25;11:592656. doi: 10.3389/fimmu.2020.592656 (PMC7723833; doi:10.3389/fimmu.2020.592656)
Supplement: Supplementary file 2 [file Table_1.pdf]

Supplemental Table 1. List of Antibodies used in the study

Antibody*	Clone	Conjugate
CD3	OKT3	APC
CD4	RPA-T4	PerCP-Cy5.5
CD8α	SK1	FITC
CD8α	SK1	PerCP-Cy5.5
CD8β	SIDI8BEE	PE-Cy7
CD14	63D3	FITC
CD16	3G8	PerCP
CD19	HIB19	FITC
CD28	CD28.2	PE
CD45RA	HI100	FITC
CD56	HCD56	PE
CD202b (Tie2/Tek)	33.1(Ab33)	Alexa Fluor 647
CCR7 (CD197)	G043H7	PE
NKG2D (CD314)	1D11	PE
KIR2DL1 (CD158a)	HP-MA4	PE
IFNɣ	4S.B3	FITC
Mouse IgG1	MOPC-21	FITC

* All antibodies but CD8β (from eBioscience) were from Biolegend
